# Exogenous Spermidine and Amino-Ethoxyvinylglycine Improve Nutritional Quality via Increasing Amino Acids in Rice Grains

**DOI:** 10.3390/plants13020316

**Published:** 2024-01-20

**Authors:** Ying Liu, Yi Jiang, Xiaohan Zhong, Chaoqing Li, Yunji Xu, Kuanyu Zhu, Weilu Wang, Junfei Gu, Hao Zhang, Zhiqin Wang, Lijun Liu, Jianhua Zhang, Weiyang Zhang, Jianchang Yang

**Affiliations:** 1 Jiangsu Key Laboratory of Crop Genetics and Physiology/Jiangsu Key Laboratory of Crop Cultivation and Physiology, Agricultural College, Yangzhou University, Yangzhou 225009, China; mx120210744@stu.yzu.edu.cn (Y.L.); ljliu@yzu.edu.cn (L.L.); 2 Jiangsu Co-Innovation Center for Modern Production Technology of Grain Crops, Yangzhou University, Yangzhou 225009, China; 3 Joint International Research Laboratory of Agriculture and Agri-Product Safety, Yangzhou University, Yangzhou 225009, China; yunjixu@yzu.edu.cn (Y.X.);; 4 Department of Biology, Hong Kong Baptist University, Hong Kong 999077, China; jzhang@hkbu.edu.hk; 5 The State Key Laboratory of Agrobiotechnology, The Chinese University of Hong Kong, Hong Kong 999077, China

**Keywords:** amino acid, polyamine, ethylene, rice, superior grains, inferior grains

## Abstract

Polyamines and ethylene are key regulators of the growth and development, quality formation, and stress response of cereal crops such as rice. However, it remains unclear whether the application of these regulators could improve the nutritional quality via increasing amino acids in rice grains. This study examined the role of exogenous polyamines and ethylene in regulating amino acid levels in the milled rice of earlier-flowered superior grain (SG) and later-flowered inferior grain (IG). Two rice varieties were field grown, and either 1 mmol L^−1^ spermidine (Spd) or 50 μmol L^−1^ amino-ethoxyvinylglycine (AVG) was applied to panicles at the early grain-filling stage. The control check (CK) was applied with deionized water. The results showed that the Spd or AVG applications significantly increased polyamine (spermine (Spm) and Spd) contents and decreased ethylene levels in both SG and IG and significantly increased amino acid levels in the milled rice of SG and IG relative to the CK. Collectively, the application of Spd or AVG can increase amino acid-based nutritional quality and grain yield via increasing polyamine (Spm and Spd) contents and reducing ethylene levels in both SG and IG of rice.

## 1. Introduction

Rice (*Oryza sativa* L.) is a major staple crop worldwide [1]. Amino acids in rice grains are very important nutrients for humans. There are 20 main amino acids in the ripe grain (caryopsis) of rice, including eight essential amino acids (EAAs) and twelve non-essential amino acids (NEAAs) [2,3]. The level of amino acids is a crucial determinant of the nutritional quality of rice and therefore plays a fundamental role in supporting healthy and sustainable human diets and food systems [4,5].

Grains on a rice panicle can usually be divided into two types: superior grain (SG) and inferior grain (IG) [6,7,8]. Compared to the late-flowered IG located at the proximal part of a panicle, the SG located at the apical part of a panicle usually flowers earlier, fills faster, and has a larger weight [6,7,8]. SG and IG grain-filling characteristics and mechanisms have been widely studied [6,7,8]. It is not known, however, whether there is a difference in amino acid anabolism between the two types of grains and the underlying mechanism.

Polyamine (PA) and ethylene play pivotal roles in higher plants such as rice by serving as crucial mediators of essential processes, including cell division, seed development, growth and development, senescence, and responses to environmental stresses [9,10,11]. PA in higher plants comprises putrescine (Put), spermidine (Spd), and spermine (Spm). The majority of these polyamines exist as free forms, accounting for over 80% of the total polyamines [12,13,14]. PA (especially Spd and Spm) plays a positive role in enhancing grain-filling to increase the grain yields of cereal crops such as rice, wheat, and maize [15,16,17,18]. Reports indicate that an increase in the content of Spd and Spm is accompanied by a higher amino acid level in milled rice grains [13]. These findings suggest that PA may play a crucial role in regulating the biosynthesis of amino acids in rice grains. In contrast to PA, higher levels of ethylene and 1-aminocylopropane-1-carboxylic acid (ACC; a direct precursor of ethylene biosynthesis) can inhibit grain-filling and amino acid biosynthesis in rice grains [13,19,20,21]. Extensive research has explored amino acid content and composition in mature rice grains, as well as the links to cultivation practices and environmental factors [22,23,24]. However, there is insufficient information regarding the influence of exogenous Spd and aminoethoxyvinylglycine (AVG), which inhibits ethylene synthesis by blocking ACC synthesis, on the amino acid levels in both SG and IG of rice.

The objective of this study was to examine whether and how Spd and AVG applications regulate amino acid levels in SG and IG of rice. To gain insight into the physiological mechanism underlying these biological processes, the levels of EAAs and NEAAs, as well as the activities of key enzymes involved in PA biosynthesis or metabolism, were assayed in SG and IG of two rice varieties under Spd and AVG applications. This study will provide valuable insights into strategies for improving the amino acid levels, nutritional quality, and grain yield in rice.

## 2. Materials and Methods

### 2.1. Plant Materials and Treatments

**Figure 1:**
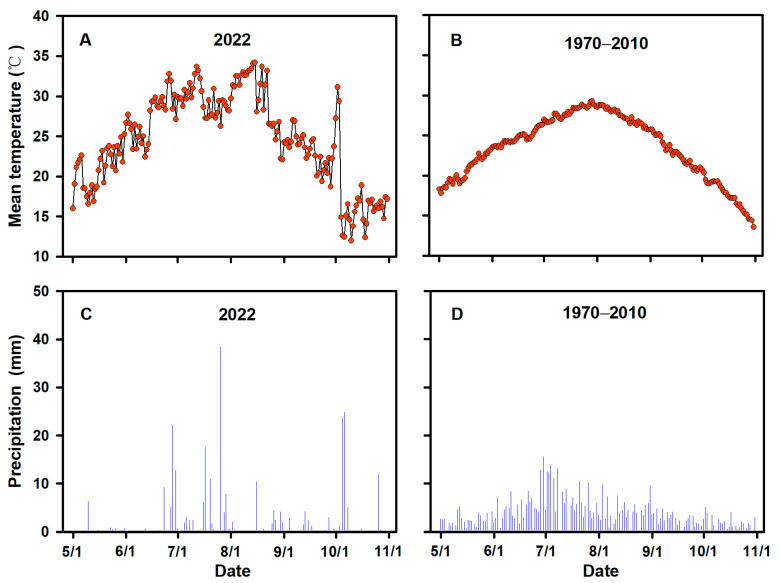
Mean temperature and precipitation in 2022 (**A**,**C**) and from 1970 to 2010 (**B**,**D**) during the rice growing period (May to October) close to the experimental site.

To ensure the accuracy and reliability of this study, we selected two representative rice varieties, namely Yangdao 6 (YD–6, *indica*) and Jinxiangyu 1 (JXY–1, *japonica*). The two varieties are widely cultivated in local rice production because of their high grain yield. Both rice varieties (YD–6 and JXY–1) were grown at the experiment farm of Yangzhou University, Jiangsu Province, China (32.30′ N, 119.25′ E, 21 m altitude) during the rice growing season (May to October) in 2022. The type of soil in the field was a sandy loam (Typic Fluvaquent, Entisol, US classification) and the soil composition was as described by Zhang et al. [25]. The seeds of the two rice varieties (YD–6 and JXY–1) were purchased from Jiangsu Kingearth Seed CO., Ltd., Yangzhou, China. The mean temperature and precipitations in 2022 and the average values of annual temperature and precipitation from 1970 to 2010 during the rice growth period (May to October) close to the experimental site are shown in Figure 1.

The seedlings of YD–6 and JXY–1 were field-grown on 15 May 2022 and manually transplanted into a paddy field with a hill spacing of 0.25 m × 0.16 m, with two seedlings per hill after a 25–day period, in accordance with local practices to achieve high productivity. Each rice variety had three independent plots as replicates, and each plot size was 4.5 m × 5.0 m. Nitrogen (200 kg ha^−1^) in urea form was applied, with 40% at 1 day before transplanting, 20% at 7 days after transplanting, 20% at panicle initiation (leaf remainder: 4.0–3.5), and 20% at spikelet differentiation (leaf remainder: 2.0–1.6). The synchronous relationship between leaf remainder and the development process of rice was described by Ling et al. [26]. Apart from draining the soil water at the end of tillering and one week prior to the final harvest, the water level was maintained at 1–2 cm, following recommended farming practices.

### 2.2. Spd and AVG Application

Two rice varieties (YD–6 and JXY–1) grown in the abovementioned plots were used for this chemical treatment experiment. Two chemical reagents, including 1 mmol L^−1^ Spd and 50 μmol L^−1^ AVG, were applied to the panicles of YD–6 and JXY–1 at 7 days post-anthesis using a paintbrush. Control check (CK) was applied with the same volume of deionized water. Each of the chemical reagents (500 mL per m^2^) was applied daily for 5 days to ensure practical treatment effect, and applied in an area of 6 m^2^ in each plot of each rice variety, with three independent replications.

### 2.3. Sampling 

One hundred panicles of the YD-6 and JXY-1 were sampled from the area of chemical treatments in each plot at 10 and 20 days after spraying chemical reagents. The SG, which flowered on the first 2 days (located on apical primary branches) within a panicle, and IG, (located on proximal secondary branches) which flowered on the last 2 days within a panicle, were separated from the sampled panicles. Some fresh samples of SG and IG were used to assay ethylene release rate. Remaining samples were frozen in liquid nitrogen for 2 min and then stored at −80 °C for determination of the contents of EAAs and NEAAs, Spm, Spd, Put, and ACC, as well as activities of ADC, SAMDC, and PAO, which were involved in PA synthesis or metabolism in both SG and IG.

### 2.4. Determination of PA Contents

Free PA (Put, Spd, and Spm) in SG and IG of YD–6 and JXY–1 (10 and 20 days after spraying chemical reagents) were extracted and purified according to the methods of Flores and Galston [27] with some modification. Separately, the SG (0.5–1.0 g) and IG (0.5–1.0 g) were thoroughly mixed in a pre-chilled mortar and pestle with 3–5 mL of 5% (*v*/*v*) perchloric acid. The homogenate was left to incubate at 4 °C for 2 h, then centrifuged at 15,000× *g* for 20 min. After centrifugation, the supernatant was collected. A 1 mL aliquot of the supernatant and standard solutions of Put, Spd, and Spm were then derivatized at 37 °C for 1 h using 20 μL of benzoyl chloride. The reaction was terminated by adding 2 mL saturated sodium chloride. After adding 2 mL diethyl ether to the solution, the mixture was centrifuged to remove the diethyl ether layer. After air drying, PA (Put, Spd, and Spm) was quantified following the method of Islam et al. [11] by using high-performance liquid chromatography (Waters 2695 Separations Module; Waters, Milford, MA, USA). PA (Put, Spd, and Spm) contents in both SG and IG of rice were expressed as nmol g^−1^ dry weight (DW).

### 2.5. Determination of Arginine Decarboxylase (ADC), S-Adenosylmethionine (SAMDC) and Polyamine Oxidase (PAO) Activities

Frozen SG (0.2 g) and IG (0.2 g) of YD–6 and JXY–1 (10 and 20 days after spraying chemical reagents) were extracted with 0.05 mol L^−1^ HCl, pH 7.4 phosphate buffer saline, respectively, and then centrifuged at 3000× *r* for 10–15 min. The supernatant was used to determine the activity of ADC, SAMDC, and PAO, which were assayed by using the ELISA method with an assay kit (Comin Biotechnology Co., Ltd., Suzhou, China) according to the manufacturer’s instructions. The activities of ADC, SAMDC, and PAO in SG and IG were expressed in unit g^−1^ h^−1^ fresh weight (FW).

### 2.6. Determination of Ethylene Release Rate and ACC Content

Ethylene release rate from SG and IG of YD–6 and JXY–1 was measured according to the methods of Zhao et al. [28], with modifications. Briefly, separately sampled SG and IG (10 and 20 days after spraying chemical reagents) were placed between two moist paper sheets for 1 h at 27 °C in the dark, allowing the wound ethylene to subside. Each sample containing 2.0–5.0 g of SG and IG was subsequently transferred to 20 mL glass vials containing moist filter paper. They were immediately sealed with airtight SubaSeal stoppers and incubated at 27 °C in darkness for 6 h. A 1 mL gas sample was withdrawn through the stopper with a gas-tight syringe and EER was determined using gas chromatography (HP5890 Series II, Hewlett Packard, Palo Alto, CA, USA). The ACC content in SG and IG of rice (10 and 20 days after spraying chemical reagents) was quantified following the method of Faroza et al. [29], and the production of ethylene from ACC was determined using the abovementioned gas chromatography. The ethylene release rate and the ACC contents in SG and IG of rice were expressed as nmol g^−1^ DW h^−1^ and nmol g^−1^ DW, respectively.

### 2.7. Determination of Hydrolyzed Amino Acid Content and Yield

The method for determining hydrolyzed amino acid contents in milled rice of SG and IG of YD–6 and JXY–1 was recommended by Mirtaleb et al. [30] and Mossé et al. [31], by using an automatic amino acid analyzer (Biochrom 30; Biochrom, Cambridge, UK). In this study, the method used by Wu et al. [32] was employed to classify the total amino acids into EAAs, including Methionine (Met), Lysine (Lys), Threonine (Thr), Isoleucine (Ile), Valine (Val), Phenylalanine (Phe), and Leucine (Leu), as well as NEAAs, including Glutamate (Glu), Aspartic acid (Asp), Arginine (Arg), Alanine (Ala), Proline (Pro), Tyrosine (Tyr), Glycine (Gly), Serine (Ser), Histidine (His), and Cysteine (Cys).

Amino acid yield (mg grain^−1^) = amino acid content (mg g^−1^ DW) × grain weight (mg) × 10^–3^ (1)

### 2.8. Final Harvest

The determination of grain yield and yield components was carried out using the method of Yoshida et al. [33]. Grain yield of YD–6 and JXY–1 was determined from a harvest area of 3 m^2^ in each chemical-treatment plot (excluding borders) and adjusted to 14% moisture in the study year. The yield components, including panicle number per square meter, spikelet number per panicle, percentage of fully filled grain, and 1000–grain weight, were determined from randomly sampled plants within a 0.8 m^2^ area (excluding the borders) from each plot. The percentage of fully filled grains was defined as fully filled grains with specific gravity ≥ 1.06 g cm^–3^ as a percentage of total spikelets.

### 2.9. Statistical Analysis

Analysis of variance was performed using SPSS software (IBM SPSS Statistics for Windows, Version 26.0., Armonk, NY, USA). Data from each sampling date were analyzed separately, and average values were tested by least significant difference at *p* < 0.05 (LSD_0.05_).

SigmaPlot software (Version 10.0, Systat Software Inc., San Jose, CA, USA) was used to construct the bar charts. R software (Corrplot, version 4.1.1, https://cran.r-project.org, accessed on 1 October 2023) was used to evaluate the correlations of PA and ACC contents, ethylene release rate, and activities of ADC, SAMDC, and PAO with the amino acid content and yield in milled rice, and constructed the correlation heatmap.

## 3. Results

### 3.1. Grain Yield

**Figure 2:**
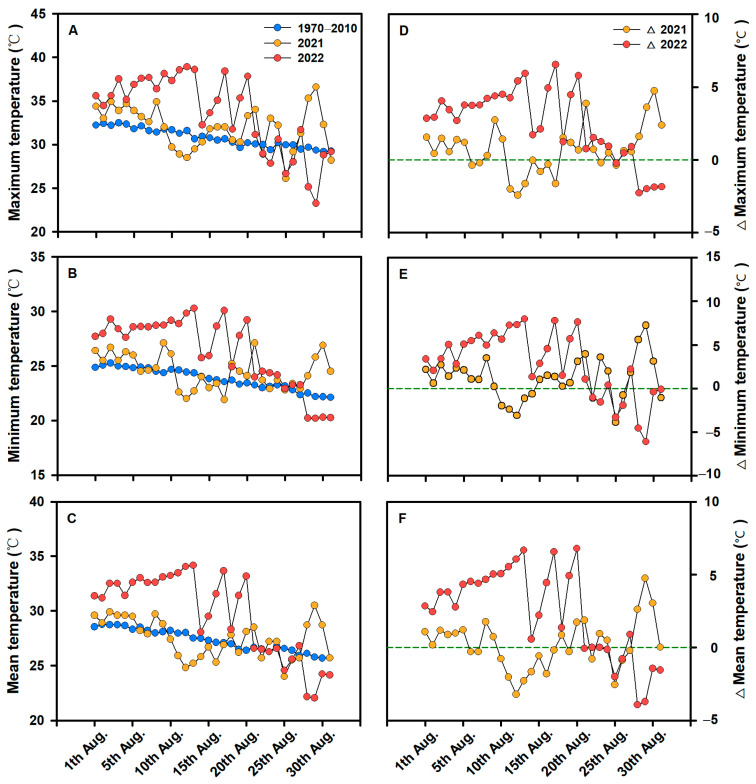
Maximum (**A**), minimum (**B**), and mean temperature (**C**) in August from 1970 to 2010, 2021, and 2022, as well as the changes in maximum (**D**), minimum (**E**), and mean temperature (**F**) in August 2021 and 2022 relative to the temperature from 1970 to 2010. ∆ indicates the extent of change in the maximum, minimum, and mean temperature in August 2021 and 2022 relative to the temperature from 1970 to 2010.

The applications of Spd and AVG significantly increased the percentage of fully filled grain, grain weight, and grain yield, whereas had no significant effects on the panicle number and spikelet number per panicle of the two rice varieties because of the chemical treatments were applied at the early grain filling stage (Table 1). In August 2022, the experimental site (Yangzhou) experienced extremely high temperatures during the meiosis, flowering, and pollination period of the two rice varieties (JXY–1 and YD–6) (Figure 2), leading to a significant decrease in the percentage of fully filled grains and grain yield of the two rice varieties by 28.3–29.2% and 31.5–32.4%, respectively, compared to those of a normal year (2021) [34,35].

### 3.2. Amino Acid Content

**Figure 3:**
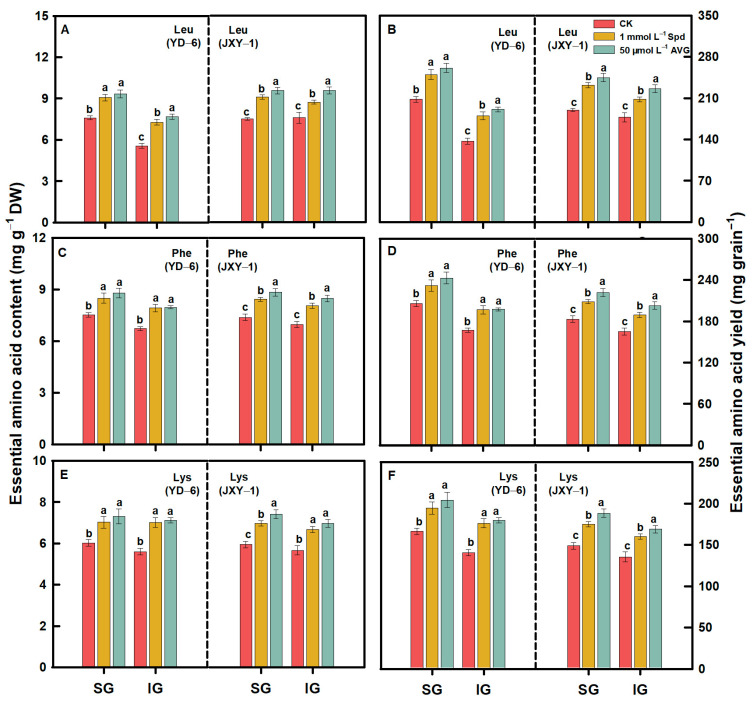
EAA (Leu (**A**,**B**), Phe (**C**,**D**), and Lys (**E**,**F**)) contents and yields in milled rice of SG and IG of YD–6 and JXY–1 under Spd and AVG applications. Amino acid yield (mg grain^−1^) = amino acid content (mg g^−1^ DW) × grain weight (mg) × 10^–3^; Leu, Leucine; Phe, Phenylalanine; Lys, Lysine. The statistical significance at the *p* = 0.05 level among the various chemical application treatments is represented by distinct lowercase letters assigned to the average values ± SD (*n* = 3) of each treatment within the identical grain type and rice variety.

**Figure 4:**
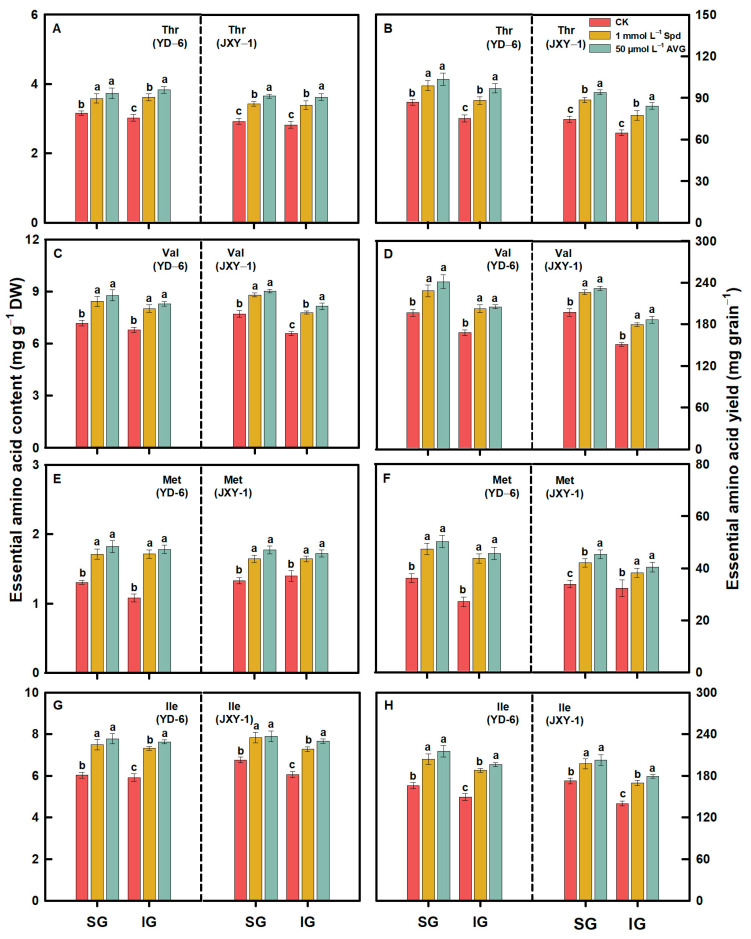
EAA (Thr (**A**,**B**), Val (**C**,**D**), Met (**E**,**F**), and Ile (**G**,**H**)) contents and yields in milled rice of SG and IG of YD–6 and JXY–1 under Spd and AVG applications. Amino acid yield (mg grain^−1^) = amino acid content (mg g^−1^ DW) × grain weight (mg) × 10^–3^; Thr, Threonine; Val, Valine; Met, Methionine; Ile, Isoleucine. The statistical significance at the *p* = 0.05 level among the various chemical application treatments is represented by distinct lowercase letters assigned to the average values ± SD (*n* = 3) of each treatment within the identical grain type and rice variety.

**Figure 5:**
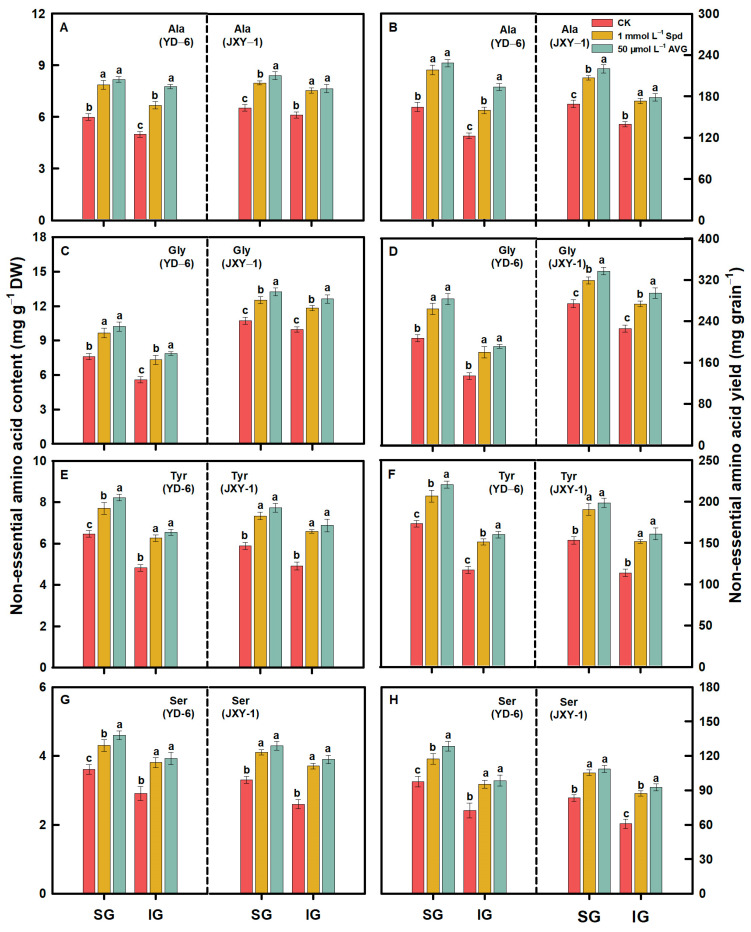
NEAA (Ala (**A**,**B**), Gly (**C**,**D**), Tyr (**E**,**F**), and Ser (**G**,**H**)) contents and yields in milled rice of SG and IG of YD–6 and JXY–1 under Spd and AVG applications. Amino acid yield (mg grain^−1^) = amino acid content (mg g^−1^ DW) × grain weight (mg) × 10^–3^; Ala, Alanine; Gly, Glycine; Tyr, Tyrosine; Ser, Serine. The statistical significance at the *p* = 0.05 level among the various chemical application treatments is represented by distinct lowercase letters assigned to the average values ± SD (*n* = 3) of each treatment within the identical grain type and rice variety.

**Figure 6:**
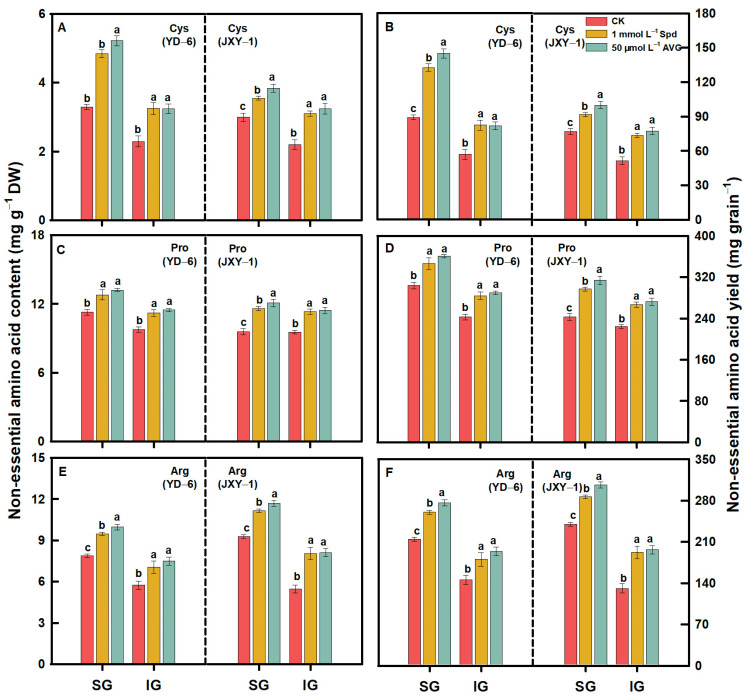
NEAA (Cys (**A**,**B**), Pro (**C**,**D**), and Arg(**E**,**F**)) contents and yields in milled rice of SG and IG of YD–6 and JXY–1 under Spd and AVG applications. Amino acid yield (mg grain^−1^) = amino acid content (mg g^−1^ DW) × grain weight (mg) × 10^–3^; Cys, Cysteine; Pro, Proline; Arg, Arginine. The statistical significance at the *p* = 0.05 level among the various chemical application treatments is represented by distinct lowercase letters assigned to the average values ± SD (*n* = 3) of each treatment within the identical grain type and rice variety.

**Figure 7:**
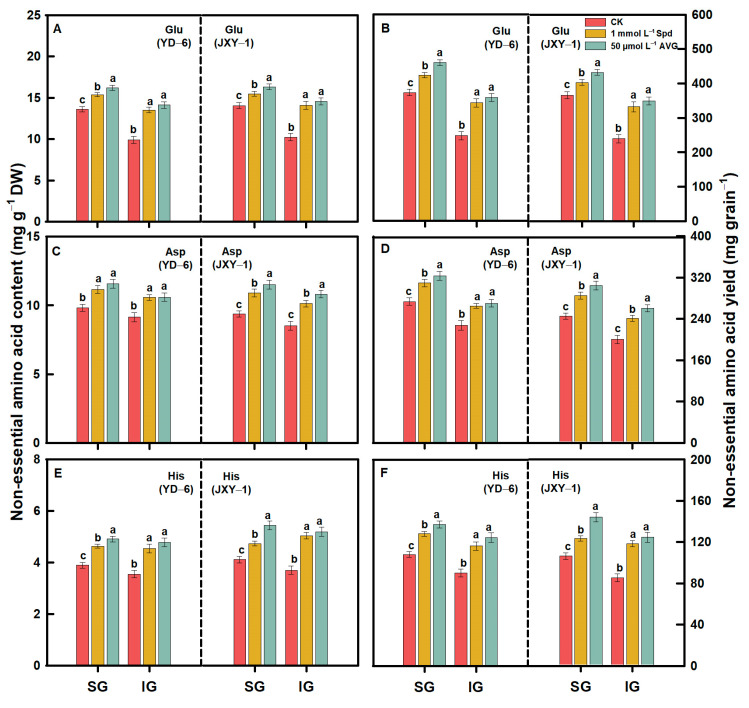
NEAA (Glu (**A**,**B**), Asp (**C**,**D**), and His (**E**,**F**)) contents and yields in milled rice of SG and IG of YD–6 and JXY–1 under Spd and AVG applications. Amino acid yield (mg grain^−1^) = amino acid content (mg g^−1^ DW) × grain weight (mg) × 10^–3^; Glu, Glutamic acid; Asp, Aspartic acid; His, Histidine. The statistical significance at the *p* = 0.05 level among the various chemical application treatments is represented by distinct lowercase letters assigned to the average values ± SD (*n* = 3) of each treatment within the identical grain type and rice variety.

Compared to the CK, spraying Spd or AVG significantly increased the contents and yields (content × grain weight) of EAAs (Met, Lys, Thr, Ile, Val, Phe, and Leu) and NEAAs (Glu, Asp, Arg, Ala, Pro, Tyr, Gly, Ser, His, and Cys) in the milled rice of SG and IG at maturity. Additionally, the application of AVG had a more pronounced effect on increasing the contents and yields of both EAAs and NEAAs in the milled rice of SG and IG of the two rice varieties. Application of Spd or AVG at the early grain filling stage could effectively alter the amino acid content in SG and IG of rice (Figure 3, Figure 4, Figure 5, Figure 6 and Figure 7).

### 3.3. PA Content

Compared to the CK, the application of both Spd and AVG led to a statistically significant increase in the contents of Spm and Spd in both SG and IG of the YD–6 and JXY–1 varieties. However, there was no significant difference in the Spm and Spd levels between Spd and AVG treatments. Furthermore, the levels of Put were the lowest when AVG was applied, while there was no significant difference between the Spd application and the CK (Table 2).

### 3.4. Ethylene Release Rate and ACC Content

The Spd and AVG applications resulted in a significant reduction in the ethylene release rate and ACC content in both SG and IG of the YD–6 and JXY–1. Notably, the Spd application displayed a significantly higher ethylene release rate and ACC content compared to the AVG application (Table 3).

### 3.5. Activities of ADC, SAMDC and PAO

The activities of ADC, SAMDC, and PAO were significantly higher during the spraying of Spd and AVG than those during CK, whereas they mostly displayed no significant difference between Spd and AVG application treatments in either SG or IG of the YD–6 and JXY–1. In general, the extent of increase in ADC and SAMDC activities under AVG and Spd application treatments was significantly higher than that of PAO activity in both SG and IG of the YD–6 and JXY–1 (Table 4).

### 3.6. Relationship of PA and ACC Contents, Ethylene Release Rate and Activities of ADC, SAMDC, and PAO with the Amino Acid Content and Yield in Milled Rice

**Figure 8:**
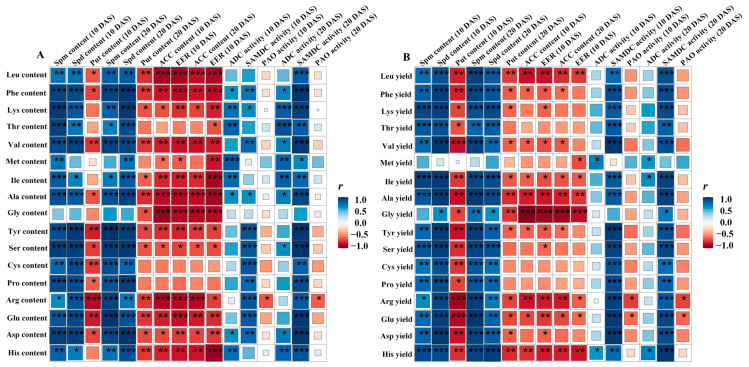
Correlations of PA and ACC contents; ethylene release rate; and activities of ADC, SAMDC, and PAO with the amino acid content (**A**) and yield (**B**) in milled rice. *, **, and *** denote statistical significance at the *p* < 0.05, *p* < 0.01, and *p* < 0.001 levels, in that order. DAS, days after spraying.

The ADC and SAMDC activities and the contents of Spd and Spm were significantly and positively correlated with EAA and NEAA contents and yields in milled rice of both SG and IG of YD–6 and JXY–1. Conversely, the ethylene release rate, ACC content, and Put content were negatively correlated with the EAA and NEAA contents and yields in milled rice of both SG and IG of YD–6 and JXY–1. Generally, there was no significant negative correlation between PAO activity and the contents and yields of EAAs and NEAAs in milled rice of both SG and IG of YD–6 and JXY–1 (Figure 8).

## 4. Discussion

Prior to this study, limited information was accessible regarding the response of amino acids, including EAA and NEAA levels in milled rice of SG and IG to Spd and AVG applications. The result herein showed that the application of Spd and AVG effectively increased the PA (Spd and Spm) contents, significantly reduced the ACC content and ethylene release rate, and synergistically increased EAA and NEAA levels in the milled rice and grain weight of SG and IG, as well as grain yield when compared with the CK. These results imply that Spd and AVG applications, by enhancing endogenous PA and reducing ethylene levels, play a positive role in enhancing grain yield and the amino acid-based nutritional quality of rice (Table 1 and Table 3, Figure 3, Figure 4, Figure 5, Figure 6 and Figure 7).

**Figure 9:**
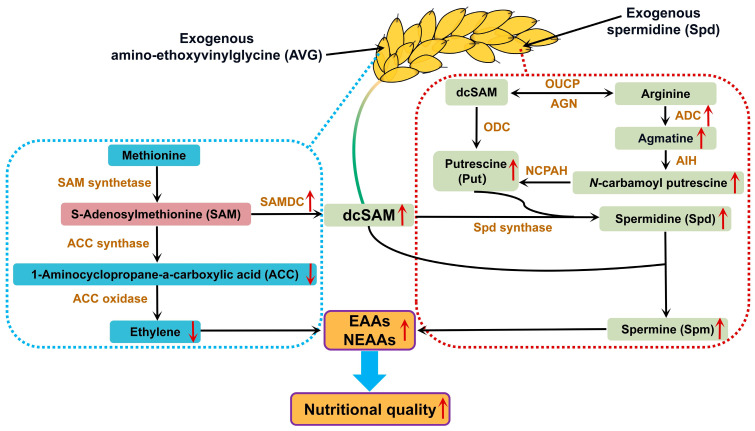
Proposed model of exogenous spermidine and amino-ethoxyvinylglycine improves the nutritional quality via increasing amino acids in rice grains. Spraying Spd and AVG can increase the activities of ADC and SAMAC, thereby increasing the synthesis of Spd and Spm, reducing the ethylene synthesis, and ultimately improving amino acid-based nutritional quality in rice. SAMDC, s-adenosylmethionine decarboxylase; dcSAM, decarboxylated s-adenosylmethionine; ODC, ornithine decarboxylase; NCPAH, *N*-carbamoylputrescine amidohydrolase; AIH, agmatine iminohydrolase; ADC, arginine decarboxylase; OUCP, ornithine-urea cycle complex; AGN, arginase. The red arrow “↑” indicates an increase, while the red arrow “↓” indicates a decrease.

The mechanism underlying Spd and AVG applications increasing amino acid levels in milled rice of SG and IG is not well understood. It is proposed that amino acid levels in grains of cereal crops could be associated with endogenous PA and ethylene levels [13,16,20,36]. In a biosynthesis pathway, PA (Spd and Spm) and ethylene share the biosynthetic precursor S-adenosyl-L-methionine (SAM), and it is conceivable that an enhancement in the biosynthesis of Spd and Spm has the potential to facilitate a decline in the synthesis of ethylene [37]. SAMDC and ADC are involved in the biosynthetic pathway of PA, with SAM and ornithine as substrates, respectively [38]. The present result exhibited that the Spd and AVG applications significantly increased the SAMDC and ADC activities and PA (Spd and Spm) levels, whereas the ethylene and ACC levels were decreased in both the SG and IG (Table 2, Table 3 and Table 4). It is speculated that there may be a potential metabolic interaction/competition between PA (Spd and Spm) and ethylene. Specifically, applications of Spd and AVG may enhance the activities of SAMDC and ADC, enabling SAM to synthesize more decarboxylated s-adenosylmethionine (dcSAM) instead of ACC. As a result, such an event can lead to an increase in Spd and Spm contents and a decrease in ethylene levels, thereby improving EAA and NEAA levels in the milled rice of SG and IG (Figure 9).

Usually, PAO plays a crucial role in PA decomposition [39,40]; the present study observed, however, that the PAO activity showed a similar increase under Spd and AVG applications as the endogenous PA (Spd and Spm) levels (Table 4). A possible explanation is that the increase in PA synthesis strength (e.g., SAMDC and ADC activities) exceeded the increase in PA oxidation strength (e.g., PAO activity), and accordingly, it was manifested as a net increase in PA (Spd and Spm) contents under Spd and AVG applications. Further study needs to be conducted to understand the mechanism underlying the biological processes.

## 5. Conclusions

Higher PA (Spd and Spm) contents and lower ethylene levels are beneficial in increasing the levels of EAAs and NEAAs in milled rice of SG and IG. The application of Spd or AVG can effectively increase PA biosynthesis, leading to increases in PA (Spd and Spm) contents and a reduction in the ethylene release rate and ACC content in both SG and IG of rice. As a result, the levels of EAAs and NEAAs in milled rice of both SG and IG are increased, thereby improving the amino acid-based nutritional quality and grain yield of rice. Notably, these data were obtained under the conditions of extremely high temperatures in August 2022, and the performance of Spd and AVG may differ as conditions change. Therefore, further investigation is needed to confirm the results and to deeply understand the mechanism underlying the biological process in which Spd and AVG regulate amino acid-based nutrient quality in rice.

## Figures and Tables

**Table 1:** Grain yield and its components of Yangdao 6 (YD-6) and Jinxiangyu 1 (JXY-1) under spermidine (Spd) and amino-ethoxyvinylglycine (AVG) applications.

Cultivar	Chemical Treatment	Panicle Number (m^−2^)	Spikelet Number Per Panicle	Fully Filled Grain (%)	1000–Grain Weight (g)	Grain Yield (g m^−2^)
YD–6	CK	218 a	143 a	62.9 b	30.1 b	619 b
	1 mmol L^−1^ Spd	220 a	145 a	67.9 a	32.8 a	689 a
	50 μmol L^−1^ AVG	219 a	145 a	68.2 a	32.5 a	694 a
JXY–1	CK	276 a	131 a	62.1 b	26.2 b	610 b
	1 mmol L^−1^ Spd	277 a	131 a	66.8 a	29.3 a	683 a
	50 μmol L^−1^ AVG	279 a	132 a	67.1 a	28.9 a	678 a

The statistical significance at the *p* = 0.05 level among the various chemical application treatments is represented by distinct lowercase letters assigned to the average values (*n* = 3) of each treatment within the identical column and rice variety.

**Table 2:** Contents of Spm, Spd, and Put (nmol g^−1^ DW) in SG and IG of YD–6 and JXY–1 under Spd and AVG application treatments.

Cultivar	Chemical Treatment	10 Days after Spraying						20 Days after Spraying					
		SG			IG			SG			IG		
		Spm	Spd	Put	Spm	Spd	Put	Spm	Spd	Put	Spm	Spd	Put
YD-6	CK	1.20 b	1.54 b	6.81 a	1.03 b	1.14 b	9.28 a	3.25 b	3.19 b	4.43 a	2.13 b	2.83 b	4.99 a
	1 mmol L^−1^ Spd	1.50 a	2.26 a	6.72 a	1.43 a	1.66 a	9.18 a	4.15 a	4.16 a	4.32 a	3.11 a	3.51 a	4.96 a
	50 μmol L^−1^ AVG	1.56 a	2.20 a	4.37 b	1.46 a	1.69 a	7.60 b	4.21 a	4.19 a	3.17 b	3.20 a	3.78 a	3.73 b
JXY-1	CK	1.13 b	1.43 b	6.39 a	1.03 b	1.05 b	9.30 a	3.13 b	2.89 b	4.01 a	2.14 b	2.67 b	4.62 a
	1 mmol L^−1^ Spd	1.51 a	2.19 a	6.33 a	1.38 a	1.53 a	9.14 a	4.18 a	4.04 a	3.96 a	3.37 a	3.82 a	4.60 a
	50 μmol L^−1^ AVG	1.40 a	2.20 a	3.80 b	1.41 a	1.58 a	7.43 b	4.22 a	4.10 a	2.72 b	3.28 a	3.90 a	3.28 b

Spm, spermine; Spd, spermidine; Put, putrescine. The statistical significance at the *p* = 0.05 level among the various chemical application treatments is represented by distinct lowercase letters assigned to the average values (*n* = 3) of each treatment within the identical column and rice variety.

**Table 3:** Ethylene release rate (nmol g^−1^ DW h^−1^) and ACC content (nmol g^−1^ DW) in SG and IG of YD–6 and JXY–1 under Spd and AVG application treatments.

Cultivar	Chemical Treatment	10 Days after Spraying				20 Days after Spraying			
		SG		IG		SG		IG	
		ACC	ERR	ACC	ERR	ACC	ERR	ACC	ERR
YD–6	CK	132 a	4.47 a	149 a	6.75 a	120 a	3.10 a	132 a	3.50 a
	1 mmol L^−1^ Spd	97.4 b	3.89 b	134 b	4.35 b	102 b	2.23 b	120 b	2.73 b
	50 μmol L^−1^ AVG	83.5 c	3.10 c	122 c	4.16 c	80.2 c	1.82 c	98.9 c	1.88 c
JXY–1	CK	104 a	4.06 a	118 a	4.53 a	94.0 a	2.44 a	115 a	2.74 a
	1 mmol L^−1^ Spd	66.7 b	2.08 b	99.1 b	3.84 b	71.0 b	1.65 b	100 b	1.85 b
	50 μmol L^−1^ AVG	53.3 c	1.29 c	83.6 c	3.01 c	49.1 c	0.85 c	77.9 c	1.05 c

ACC, 1–aminocyclopropane–1–carboxylic acid content; ERR, ethylene release rate. The statistical significance at the *p* = 0.05 level among the various chemical application treatments is represented by disti`nct lowercase letters assigned to the average values (*n* = 3) of each treatment within the identical column and rice variety.

**Table 4:** Activities of ADC, SAMDC, and PAO (U g^−1^ FW h^−1^) in SG and IG of YD–6 and JXY–1 under Spd and AVG application treatments.

Cultivar	Chemical Treatment	10 Days after Spraying						20 Days after Spraying					
		SG			IG			SG			IG		
		ADC	SAMDC	PAO	ADC	SAMDC	PAO	ADC	SAMDC	PAO	ADC	SAMDC	PAO
YD-6	CK	6.23 b	8.13 b	9.38 b	7.23 b	5.47 b	16.6 b	4.22 b	5.48 b	7.07 b	5.19 b	4.27 b	13.8 b
	1 mmol L^−1^ Spd	8.29 a	8.98 a	11.1 a	9.31 a	6.66 a	18.7 a	6.24 a	6.44 a	9.53 a	7.34 a	5.71 a	14.6 a
	50 μmol L^−1^ AVG	8.48 a	9.32 a	11.5 a	9.53 a	6.95 a	19.0 a	6.51 a	6.56 a	9.61 a	7.62 a	5.85 a	14.9 a
JXY-1	CK	6.19 b	7.02 b	9.27 b	6.76 b	4.02 b	16.7 b	4.79 b	5.11 b	7.95 b	4.45 b	4.05 b	13.9 b
	1 mmol L^−1^ Spd	8.18 a	8.85 a	11.0 a	9.70 a	5.85 a	18.7 a	6.54 a	6.63 a	9.00 a	7.16 a	5.90 a	15.8 a
	50 μmol L^−1^ AVG	8.34 a	9.02 a	11.3 a	9.43 a	5.92 a	19.1 a	6.58 a	6.66 a	9.35 a	7.22 a	6.12 a	16.1 a

ADC, arginine decarboxylase; SAMDC, s-adenosylmethionine; PAO, polyamine oxidase. The statistical significance at the *p* = 0.05 level among the various chemical application treatments is represented by distinct lowercase letters assigned to the average values (*n* = 3) of each treatment within the identical column and rice variety.

## Data Availability

Data are contained within the article.
